# Paroxysmal Discharges in Tissue Slices From Pediatric Epilepsy Surgery Patients: Critical Role of GABA_B_ Receptors in the Generation of Ictal Activity

**DOI:** 10.3389/fncel.2020.00054

**Published:** 2020-03-20

**Authors:** Simon Levinson, Conny H. Tran, Joshua Barry, Brett Viker, Michael S. Levine, Harry V. Vinters, Gary W. Mathern, Carlos Cepeda

**Affiliations:** ^1^IDDRC, Semel Institute for Neuroscience and Human Behavior, David Geffen School of Medicine, University of California, Los Angeles, Los Angeles, CA, United States; ^2^Section of Neuropathology, Department of Pathology and Laboratory Medicine, David Geffen School of Medicine, University of California, Los Angeles, Los Angeles, CA, United States; ^3^Department of Neurology, David Geffen School of Medicine, University of California, Los Angeles, Los Angeles, CA, United States; ^4^Department of Neurosurgery, David Geffen School of Medicine, University of California, Los Angeles, Los Angeles, CA, United States

**Keywords:** pediatric epilepsy, 4-aminopyridine, phaclofen, ictal activity, cortical dysplasia, slices

## Abstract

In the present study, we characterized the effects of bath application of the proconvulsant drug 4-aminopyridine (4-AP) alone or in combination with GABA_A_ and/or GABA_B_ receptor antagonists, in cortical dysplasia (CD type I and CD type IIa/b), tuberous sclerosis complex (TSC), and non-CD cortical tissue samples from pediatric epilepsy surgery patients. Whole-cell patch clamp recordings in current and voltage clamp modes were obtained from cortical pyramidal neurons (CPNs), interneurons, and balloon/giant cells. In pyramidal neurons, bath application of 4-AP produced an increase in spontaneous synaptic activity as well as rhythmic membrane oscillations. In current clamp mode, these oscillations were generally depolarizing or biphasic and were accompanied by increased membrane conductance. In interneurons, membrane oscillations were consistently depolarizing and accompanied by bursts of action potentials. In a subset of balloon/giant cells from CD type IIb and TSC cases, respectively, 4-AP induced very low-amplitude, slow membrane oscillations that echoed the rhythmic oscillations from pyramidal neurons and interneurons. Bicuculline reduced the amplitude of membrane oscillations induced by 4-AP, indicating that they were mediated principally by GABA_A_ receptors. 4-AP alone or in combination with bicuculline increased cortical excitability but did not induce seizure-like discharges. Ictal activity was observed in pyramidal neurons and interneurons from CD and TSC cases only when phaclofen, a GABA_B_ receptor antagonist, was added to the 4-AP and bicuculline solution. These results emphasize the critical and permissive role of GABA_B_ receptors in the transition to an ictal state in pediatric CD tissue and highlight the importance of these receptors as a potential therapeutic target in pediatric epilepsy.

## Introduction

There are two major GABA receptor subtypes in the central nervous system, type A (including the A-ρ subfamily) and type B (Olsen and Sieghart, 2009). The ionotropic GABA_A_ receptor mediates fast inhibitory neurotransmission and is preferentially localized in postsynaptic membranes. When the neurotransmitter binds the multimeric GABA_A_ receptor, it allosterically opens a chloride ion channel leading, in most cases, to membrane hyperpolarization (Olsen and Sieghart, 2009). In contrast, the metabotropic GABA_B_ receptor is a G protein-coupled receptor present at both pre- and post-synaptic membranes where it regulates neurotransmitter release and slow, prolonged inhibitory responses, respectively. GABA_B_ receptors function via multiple mechanisms including inwardly rectifying K^+^ channels, voltage-gated Ca^2+^ channels, and adenylyl cyclase, all of which result in either reduced neurotransmitter release or hyperpolarization of the neuron (Newberry and Nicoll, 1984; Thompson and Gahwiler, 1992; Bowery et al., 2002; Bettler et al., 2004; Frangaj and Fan, 2018). Reduced function of GABA_A_ receptors is traditionally thought to contribute to a breakdown in inhibitory neurotransmission. Thus, GABA_A_ receptor antagonists have been used to mimic some features of epileptic activity. However, the role of GABA_B_ receptors in epileptogenesis, especially in humans, is less well understood.

Spontaneous paroxysmal discharges, ictal or interictal, are rarely observed in *ex vivo* slices from pediatric or adult epilepsy surgery tissue samples resected for the treatment of pharmacoresistant epilepsy. This is probably due to the elimination of long-range excitatory inputs. In our cohort of approximately 300 cases examined thus far (Cepeda et al., 2003, 2005a,b, 2006, 2012; Abdijadid et al., 2015), epileptic activity in the form of paroxysmal depolarizing shifts or spontaneous bursting was only seen in about 2% of cases and ictal activity was never recorded in cortical pyramidal neurons (CPNs). However, fast-spiking interneurons were more prone to display ictal-like activity (Cepeda et al., 2019). Also, cytomegalic interneurons in cases of severe cortical dysplasia (CD), i.e., CD type II, may generate spontaneous paroxysmal depolarizations (Andre et al., 2007). In order to induce paroxysmal discharges in tissue slices, the ionic concentrations of the bathing solution have been manipulated (e.g., Mg^2+^ removal or increased K^+^ concentration), or proconvulsant agents such as GABA_A_ receptor antagonists (bicuculline, BIC, or picrotoxin) and K^+^-channel blockers [4-aminopyridine (4-AP)] have been used (Avoli et al., 1999, 2003; D’Antuono et al., 2004; Avoli and Jefferys, 2016). Of particular interest is the fact that human CD tissue is exquisitely sensitive to the proconvulsant effects of 4-AP, as this drug induces spontaneous seizures in about 50% of slices from CD, but not from mesial temporal lobe epilepsy cases (Avoli et al., 1999; D’Antuono et al., 2004).

4-Aminopyridine is an isomeric amine of pyridine and has been widely used to characterize K^+^ channel subtypes. It is a powerful epileptogenic agent that acts by blocking type A/D K^+^ channels (Traub et al., 1995; Mitterdorfer and Bean, 2002; Szente et al., 2002). It increases neurotransmitter release by prolonging action potential duration (Bostock et al., 1981) and in hippocampal and cortical tissue slices it also induces membrane oscillations which depend on synchronous activation of GABA receptors and facilitation of gap junctional current and/or permeability (Traub et al., 2001).

In the present study, the sensitivity of neocortical pyramidal and non-pyramidal neurons to proconvulsants 4-AP and BIC, alone or in conjunction, was tested in slices from tissue resected surgically for the treatment of refractory epilepsy in pediatric surgery patients. Pathologies included CD type I and type II, tuberous sclerosis complex (TSC, a genetic form of severe CD), and non-CD etiologies (e.g., perinatal stroke, tumor). Normal and abnormal cell types, including normal-appearing CPNs and interneurons, cytomegalic and immature pyramidal neurons, and balloon/giant cells were studied. In addition, we examined the modulatory effects of GABA_B_ receptors on paroxysmal discharges induced by 4-AP. Taken together, the present study supports the idea that GABA_B_ receptors play a key role in the transition from interictal to ictal activity, especially in CD cases.

## Materials and Methods

### Cohort and Standard Protocol Approvals

The Institutional Review Board at the University of California Los Angeles (UCLA) approved the use of human subjects for research purposes, and parents or responsible persons signed written informed consents and HIPAA authorizations. Children undergoing resective surgery with the UCLA Pediatric Epilepsy Program to help control their medically refractory focal epilepsy were sequentially recruited from December 2002 to October 2016. For the present study, cortical tissue samples from four groups of etiologies were included; CD type I, CD type IIa/b (Blumcke et al., 2011; Guerrini et al., 2015), TSC, and non-CD etiologies including tumor, infarct, Sturge–Weber syndrome (SWS), polymicrogyria, multicystic encephalopathy, Aicardi syndrome, and three cases with mild, undetermined cortical pathology.

### Electrocorticography and Surgical Resection

The site and margin of the surgical resection were based on recommendations from a multidisciplinary meeting after careful consideration of the presurgical evaluation of each patient, as previously described (Cepeda et al., 2003, 2005b, 2014, 2019). For the four groups of etiologies, our goal was complete resection of the epileptogenic zone primarily defined by non-invasive testing (Lerner et al., 2009; Hemb et al., 2010), including video-EEG capturing ictal events, high-resolution magnetic resonance imaging (MRI), and ^18^-fluorodeoxyglucose positron emission tomography (FDG-PET), as well as magnetic source imaging and co-registration of MRI and FDG-PET when the initial battery of tests was inconclusive (Salamon et al., 2008).

### Slice Preparation and Electrophysiological Recordings

After surgical resection, the tissue samples were immediately immersed in ice-cold artificial cerebrospinal fluid (ACSF) enriched with sucrose for better preservation and then expeditiously hand-carried out of the operating room and transported directly to the laboratory within 5–10 min. The high sucrose-based slicing solution contained (in mM): 208 sucrose, 10 glucose, 26 NaHCO_3_, 1.25 NaH_2_PO_4_, 2.5 KCl, 1.3 MgCl_2_, 8 MgSO_4_. Coronal slices (300 μm) were cut and transferred to an incubating chamber containing ACSF (in mM): 130 NaCl, 3 KCl, 1.25 NaH_2_PO_4_, 26 NaHCO_3_, 2 MgCl_2_, 2 CaCl_2_, and 10 glucose) oxygenated with 95% O_2_–5% CO_2_ (pH 7.2–7.4, osmolality 290–310 mOsm/L, 32–34°C). In selected experiments, the ACSF solution was modified to reduce the amount of glucose and introduce additional energy substrates, i.e., ketones and pyruvate, more akin to those used by developing brains (Holmgren et al., 2010; Zilberter et al., 2010). This bathing solution, also known as enriched ACSF (eACSF) contained (in mM): 130 NaCl, 3 KCl, 1.25 NaH_2_PO_4_, 26 NaHCO_3_, 2 MgCl_2_, 2 CaCl_2_, 5 glucose, 5 Na pyruvate, 2 Na 3-hydroxybutyrate (BHB). Slices were allowed to recover for an additional 60 min at room temperature prior to recording. All recordings were performed at room temperature using an upright microscope (Olympus BX51WI) equipped with infrared-differential interference contrast (IR-DIC) optics.

Whole-cell patch clamp recordings in voltage- or current-clamp modes were obtained from different cell types (layers II–V) visualized with IR-DIC (Cepeda et al., 2012). The patch pipette (3–5 MΩ resistance) contained a cesium-based internal solution (in mM): 125 Cs-methanesulfonate, 4 NaCl, 1 MgCl_2_, 5 MgATP, 9 EGTA, 8 HEPES, 1 GTP-Tris, 10 phosphocreatine, and 0.1 leupeptin (pH 7.2 with CsOH, 270–280 mOsm/L) for voltage clamp recordings. K-gluconate-based solution containing (in mM): 112.5 K-gluconate, 4 NaCl, 17.5 KCl, 0.5 CaCl_2_, 1 MgCl_2_, 5 ATP, 1 NaGTP, 5 EGTA, 10 HEPES, pH 7.2 (270–280 mOsm/L) was used for current clamp recordings. After breaking the seal, basic cell membrane properties (capacitance, input resistance, decay time constant) were recorded while holding the membrane potential (V_h_) at −70 mV. Electrode access resistances during whole-cell recordings were less than 25 MΩ (range 8–25 MΩ). Electrodes also contained 0.2% biocytin in the internal solution to label recorded cells. Proconvulsant drugs included 4-AP (100 μM), BIC (10–20 μM), and the GABA_B_ receptor antagonist phaclofen (6–25 μM). In voltage and current clamp modes, the latency, frequency, and amplitude of 4-AP oscillations were measured using the Clampfit software (v 10.3). As all slices were treated with 4-AP, alone or in combination with other drugs, the number of cells recorded is equivalent to the number of slices.

### Statistics

In the text and figures, results are expressed as mean ± SEM. For group comparisons, we used one-way ANOVA (with Bonferroni correction) or if normality failed the Kruskal–Wallis ANOVA on Ranks with pairwise multiple comparisons (Dunn or Holm–Sidak methods) was used. For simple comparisons between two groups, we used the Student’s *t*-test and for comparisons between proportions the Chi-square test was used. SigmaStat (3.5) software was used for all statistical analyses. Differences were deemed statistically significant if *p* < 0.05.

## Results

### Cohort

The number of cases included in the present study, their pathologies, and the number of cells recorded separated by type are shown, in abridged form, in Table 1. The average age of the pediatric population examined, regardless of pathology, was 3 ± 0.43 years (*n* = 80, 45 male and 35 female). There was a strong trend for CD patients to be younger than patients with TSC or non-CD pathologies, but the difference did not reach statistical significance (*p* = 0.056). Similarly, the average age of CD type I (2.5 ± 0.26 year, *n* = 18, 13 male and five female) and type II (2.4 ± 0.26 year, *n* = 28, 13 male and 15 female) cases or the TSC (3.6 ± 0.43 year, *n* = 18, 11 male and seven female) and non-CD (3.9 ± 0.35 year, *n* = 16, eight male and eight female) cases were not significantly different between them. In all cases, histopathological analyses confirmed initial clinical and imaging diagnoses. At the time of surgery, patients were taking antiepileptic drugs (AEDs) to control seizures. AEDs included topiramate, clonazepam, phenobarbital, lamotrigine, zonisamide, carbamazepine, or adrenocorticotropic hormone.

**TABLE 1:** Pediatric epilepsy cohort.

	**Age**	**Pyramidal**		**Balloon/**
**Pathology (cases)**	**(years)**	**cells**	**Interneurons**	**giant cells**
CD type I (*n* = 18)	2.9 ± 0.6	59	5	–
CD type II (*n* = 28)	2.5 ± 0.4	66	10	4
TSC (*n* = 18)	3.1 ± 0.6	45	1	6
Non-CD (*n* = 16)	3.6 ± 0.6	44	5	–
Total (*n* = 80)	3.0 ± 0.3	214	21	10

### Pathological Findings

In patients with CD type I and type II histopathological analyses confirmed changes consistent with the ILAE consensus classification of FCDs (Blumcke et al., 2011). In CD type I these included moderate to severe neuronal disorganization, heterotopic neurons in the white matter, mild neuronal crowding and, in some cases, blurring of the gray–white matter junction. In patients with CD type II, in addition to neuronal disorganization, dysmorphic, cytomegalic neurons (IIa) and balloon cells (IIb) also occurred. TSC cases displayed giant cells and dysmorphic cytomegalic neurons. Severe neuronal disorganization and heterotopic neurons in the white matter were also observed. Non-CD pathologies included; six infarct cases, two patients had a cystic infarct with gliosis, one patient had multiple cortical and subcortical white matter infarcts with marked neuronal loss, two other patients had some neuronal crowding but no evidence of dyslamination, while the remaining patient had some scattered heterotopic neurons in the white matter. The two SWS cases showed pronounced leptomeningeal angiomatosis, with predominantly thin-walled, venous channels. The tumor case had a small oligodendroglial hamartoma. Additionally, one case presented with multicystic encephalopathy, one with Aicardi syndrome without evidence of CD, and two cases presented with polymicrogyria and pachygyria. Finally, in three cases, there was no definitive histopathological diagnosis. The first two showed varying amounts of heterotopic neurons in the white matter, and the third had a right ventricular cyst and showed gliosis of the ventricular wall and nodular gray matter but no signs of dysplastic changes.

### Cell Types and Basic Membrane Properties

All cells (*n* = 245) were recorded using the whole-cell patch clamp configuration in both current clamp (*n* = 69) and voltage clamp (*n* = 176) modes. Basic membrane properties, including cell capacitance, input resistance, and decay time constant were determined in voltage clamp mode. Cells were recorded in layers II/V from frontal, parietal, and temporal areas. As expected, the most abundant cell type in our cohort was normal-appearing CPNs (*n* = 182). In CD types I and IIa/b and in TSC cases, additional cell types were observed including dysmorphic/cytomegalic pyramidal neurons (*n* = 17), immature-looking pyramidal neurons (*n* = 15), and balloon/giant cells (*n* = 4 in CD type IIb and *n* = 6 in TSC cases) (Blumcke et al., 2011; Guerrini et al., 2015). Giant cells have morphological and electrophysiological properties that are similar to those of balloon cells (Cepeda et al., 2003; Jozwiak et al., 2006; Grajkowska et al., 2008; Abdijadid et al., 2015). Balloon/giant cells appear to be enlarged astrocytes but in the cerebral cortex, they can also express neuronal markers (Cepeda et al., 2003, 2010; Jozwiak et al., 2006). A small number of interneurons were observed across the different pathologies (*n* = 21, including one cytomegalic interneuron in a CD type IIb case with associated hemimegalencephaly) (Table 1).

**FIGURE 1:**
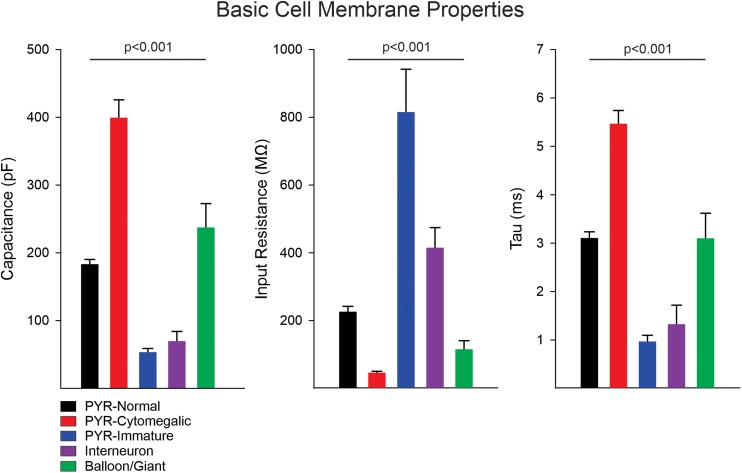
Membrane properties of the different cell types. Measurement of basic cell membrane properties (capacitance, input resistance, and time constant) in voltage clamp mode demonstrated statistically significant differences among cell groups (*p* < 0.001, one-way ANOVA on Ranks). The cell capacitance of cytomegalic pyramidal neurons was significantly larger than that of normal CPNs, immature pyramidal neurons, and interneurons (all at *p* < 0.001, pairwise multiple comparisons using Dunn’s method), whereas it was not different from that of balloon/giant cells (*p* = 0.3). Similarly, the cell capacitance of normal CPNs and balloon/giant cells was larger than that of interneurons and immature CPNs (both at *p* < 0.001). Comparison of cell capacitance between balloon/giant cells and normal CPNs or that between interneurons and immature CPNs was not significantly different. In contrast, the membrane input resistance of cytomegalic CPNs was significantly lower than that of normal and immature CPNs, as well as interneurons (all at *p* < 0.001). The input resistance of cytomegalic and normal CPNs compared with balloon/giant cells was not significantly different (both at *p* = 0.6). Immature CPNs had significantly higher input resistance compared with that of normal CPNs and balloon/giant cells (both at *p* < 0.001) but not different from that of cortical interneurons. Finally, the input resistance of interneurons was significantly higher than that of balloon/giant cells (*p* = 0.004) and normal CPNs (*p* = 0.02). The differences in time constant were similar to those in cell capacitance, except for the comparison between cytomegalic CPNs and balloon/giant cells, which was significantly lower in the latter (*p* < 0.05).

Statistically significant differences in basic cell membrane properties were observed among the different cell types (*p* < 0.001, one-way ANOVA on Ranks, see details in Figure 1 legend). In particular, normal-appearing CPNs had larger capacitance and lower input resistance than cortical interneurons. Similarly, cytomegalic CPNs had larger capacitance and lower input resistance than normal-appearing and immature CPNs. The Resting Membrane Potential (RMP) was calculated in a subset of cells recorded in current clamp mode. Significant differences were found among the main cell types (*p* = 0.001, one-way ANOVA followed by pairwise multiple comparison procedures, Holm–Sidak method). The RMP of cortical interneurons (−60 ± 1.1 mV, *n* = 12) was more depolarized than that of normal-appearing CPNs (−66.3 ± 1.2 mV, *n* = 52) (*p* = 0.02). In addition, the RMP of balloon/giant cells (−76.8 ± 3.9 mV, *n* = 5) was more hyperpolarized than that of both CPNs (*p* = 0.015) and interneurons (*p* < 0.001).

### 4-AP Oscillations in Different Cortical Cell Types in CD, TSC, and Non-CD Cases

Bath application of 4-AP (100 μM) initially increased the frequency of spontaneous glutamatergic and GABAergic synaptic events. The magnitude of this increase in frequency was calculated in a small sample of CPNs using the MiniAnalysis software (Synaptosoft). At a holding potential of −70 mV (to isolate glutamatergic events), the frequency of spontaneous glutamatergic events increased from 1.5 ± 0.3 to 5.7 ± 0.9 Hz (*p* = 0.001, *n* = 7 CPNs), whereas at + 10 mV (to isolate GABAergic events) it increased from 4.2 ± 0.5 to 8.5 ± 0.6 Hz (*p* < 0.001, *n* = 25 CPNs). After a latency of about 2–3 min 4-AP induced large-amplitude, rhythmic membrane oscillations. It has been postulated that 4-AP oscillations are the result of synchronous activation of GABA receptors and facilitation of gap junctional currents and/or permeability (Traub et al., 2001). Without ruling out completely the contribution of gap junctions, we found that 4-AP oscillations were synaptically mediated as blockade of Na^+^ channels with tetrodotoxin (TTX, 1 μM) or blockade of Ca^2+^ channels with cadmium (100 μM) abolished these membrane oscillations (Supplementary Figure S1).

Almost all CPNs and interneurons (*n* = 221) displayed 4-AP oscillations of variable frequency and amplitude. A small subset of neurons (*n* = 14) did not display oscillations or they were negligible. These cells occurred generally in the youngest cases (three CD type I, five CD type II, two TSC, and two non-CD; mean age 1.2 ± 0.3 year, range 0.3–3 year). Most were normal-appearing CPNs (*n* = 8), four were immature pyramidal neurons, and two were interneurons based on electrophysiological and morphological properties.

**FIGURE 2:**
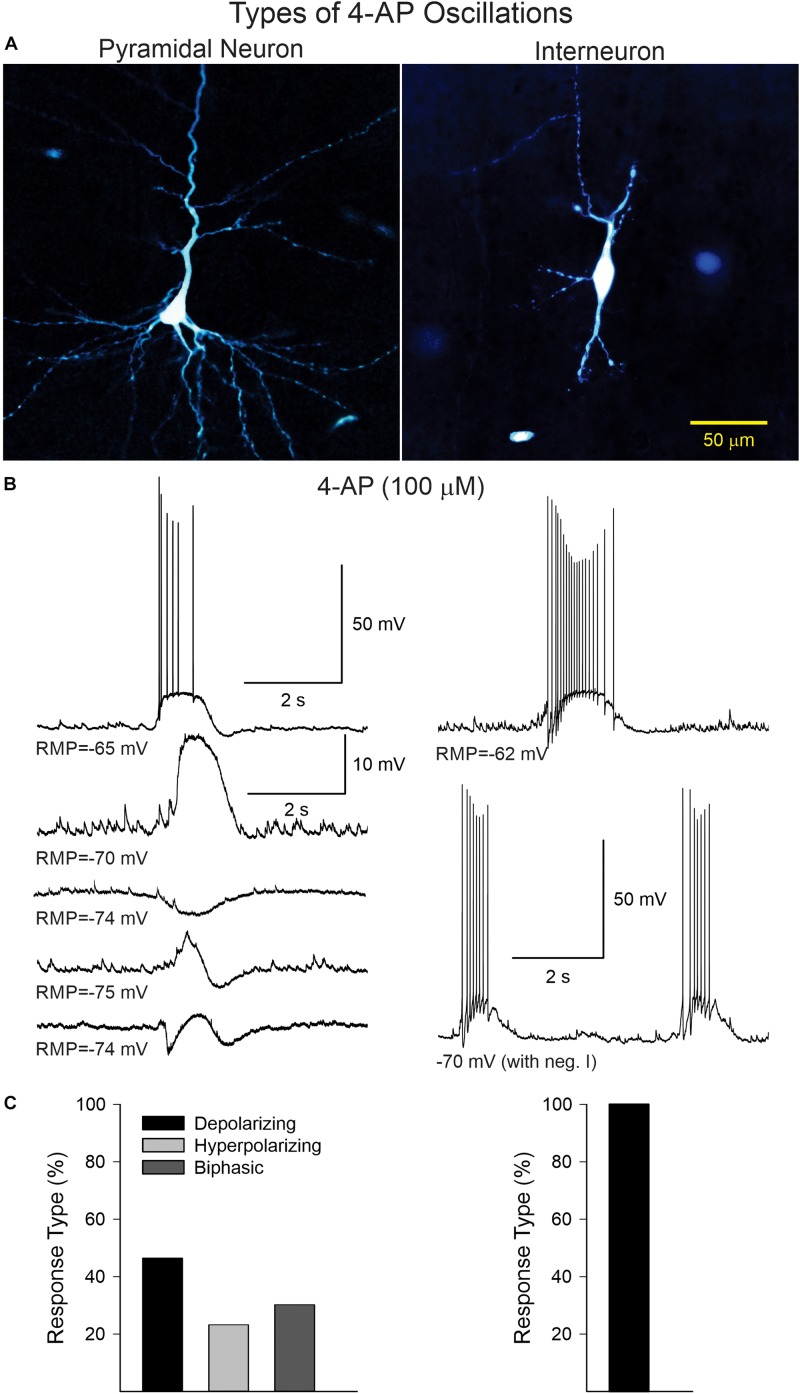
**(A)** Examples of a CPN and an interneuron recorded and filled with biocytin. **(B)** Current clamp recordings illustrating the different types of 4-AP oscillations in CPNs and interneurons. In CPNs, 4-AP induced three main types of oscillations; most were depolarizing, with or without action potentials, some were mainly hyperpolarizing, and others were biphasic (depolarization followed by hyperpolarization or vice versa). In interneurons, 4-AP consistently induced membrane depolarizations accompanied by bursts of action potentials, even at hyperpolarized potentials (with negative current). **(C)** Bar graphs show the percentage of cells displaying the different types of oscillations in CPNs and interneurons.

In current clamp mode (K-gluconate in the patch pipette), CPNs displayed three main types of 4-AP oscillations at RMP (Figure 2). Provided CPNs had an RMP more negative than the chloride reversal potential, the oscillation was purely depolarizing (*n* = 20 cells). The average depolarization amplitude was 10.8 ± 1 mV and if the depolarization was large enough it could elicit scattered action potentials. A few CPNs (*n* = 10) displayed small amplitude oscillations that were hyperpolarizing (average amplitude −5.1 ± 1 mV). At more depolarized potentials (around −60 mV) CPNs displayed biphasic responses (*n* = 13), equally divided between depolarization followed by hyperpolarization, or vice versa. In contrast, 4-AP oscillations in cortical interneurons (*n* = 13, including three FSI, nine non-FSI, and one cytomegalic interneuron) were consistently depolarizing and, in almost all cases, accompanied by bursts of action potentials. These bursts were also seen in voltage clamp mode (V_h_ = −70 mV). This suggests that 4-AP oscillations in CPNs are primarily triggered by rhythmic bursting of cortical interneurons.

In CD type IIb (*n* = 4) and TSC (*n* = 5) cases 10 balloon/giant (*n* = 4 and 6, respectively) cells were recorded. As previously described, these cells share similar morphological and electrophysiological properties including lack of inward Na^+^ or Ca^2+^ currents and no synaptic inputs but prominent K^+^ currents (Cepeda et al., 2003). We tested the effects of 4-AP on these cells. Most cells did not display any obvious effect. However, four cells (three from two CD type IIb cases and one from a TSC case) displayed very slow (3.75 ± 0.3/min), low-amplitude membrane depolarizations (2.1 ± 0.4 mV in current clamp) or inward currents (8.3 ± 2 pA in voltage clamp) (Supplementary Figure S2), suggesting they could be sensing K^+^ elevations induced by 4-AP. The function of these oscillations remains unknown, but it is possible that they may be buffering increases in K^+^ caused by 4-AP-induced paroxysmal activity, acting as a retardant of network synchrony.

### 4-AP Oscillations in CPNs and Interneurons From Different Pathologies

**FIGURE 3:**
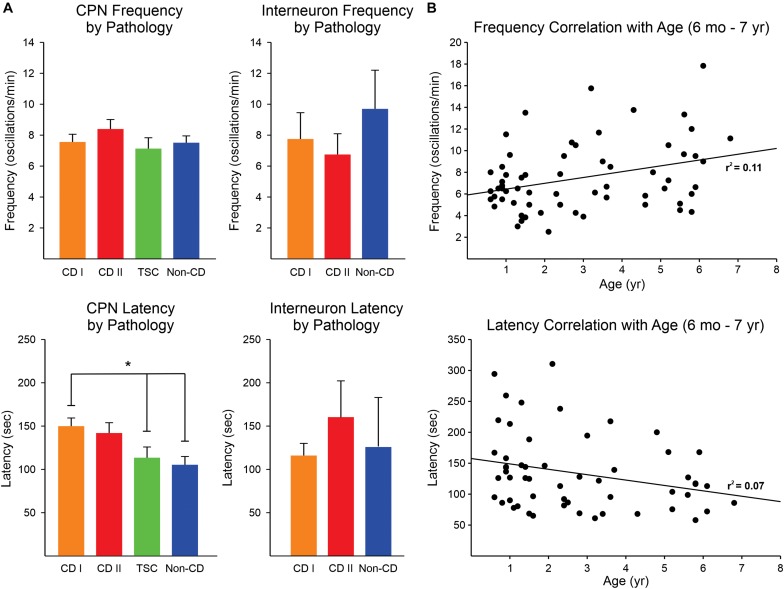
Frequency and latency of 4-AP oscillations by pathology. **(A)** No difference was observed in the frequency of 4-AP oscillations in CPNs and interneurons among pathologies, only a trend for higher frequency of oscillations in CPNs in the CD type II group (upper panels). The latency for the first occurrence of 4-AP oscillations in CPNs was significantly increased in CD type 1 cases compared with non-CD and TSC cases (lower panels) (*p* < 0.05, one-way ANOVA with Bonferroni correction). CD type II cases also had a strong trend for longer latencies. No difference in latency among groups occurred in interneurons. In TSC, only one interneuron was recorded, so this group was not included in the graphs. **(B)** Correlations between frequency and latency of 4-AP oscillations with age. There was a minimal, non-significant correlation between age and frequency/latency of 4-AP oscillations. *p < 0.05.

No significant difference was found in the frequency of 4-AP oscillations of CPNs regardless of pathology, although there was a trend for CPNs from CD type II cases to display higher frequencies (Figure 3A, upper graphs). The lowest frequencies were observed in CPNs from TSC cases. The frequency range among CPNs was between 2 and 29 oscillations/min. Cells with the highest frequencies (≥15 oscillations/min) were consistently found in CD type I (5.3%) and II (11.5%) and TSC (4.8%) cases compared with non-CD cases (2.4%). Although overall the difference in proportions among groups was not statistically significant, the difference between the CD type II and the non-CD groups almost reached statistical significance (*p* = 0.09, Chi-square test). Interestingly, the latency to the first oscillation was significantly different among groups (*p* = 0.002, Kruskal–Wallis one-way analysis of variance on Ranks). *Post hoc* pairwise multiple comparisons showed that the latency in the CD type I group was significantly longer than that of the non-CD group (*q* = 3.3) and the TSC group (*q* = 2.9) (Figure 3A, lower graphs). This suggests that network synchrony requires more time when there is cortical disorganization or as a result of the presence of abnormal cells, at least for the CD type 1 and non-CD group comparison. This does not explain, however, why the latency in the TSC group was also decreased. Finally, there was no significant correlation between frequency and latency of 4-AP oscillations with age, only a small trend for frequency to increase and latency to decrease with age (Figure 3B).

### Electrophysiological Characterization of 4-AP-Induced Membrane Oscillations in Pediatric Epilepsy Tissue Samples

**FIGURE 4:**
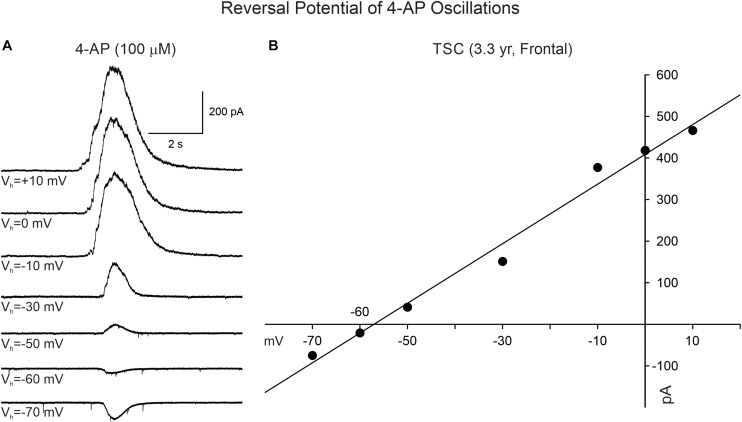
Reversal potential of 4-AP oscillations in CPNs recorded in voltage clamp mode. **(A)** Traces show voltage clamp recordings of a CPN. After 4-AP oscillations were induced, the holding potential was changed from + 10 to −70 mV. The amplitude of the 4-AP oscillation decreased from +10 to −50 mV and reversed between −60 and −70 mV. **(B)** The IV relationship of the 4-AP oscillation determined that the equilibrium potential occurred around −57 mV, corresponding to the predicted chloride equilibrium potential based on our internal patch solution.

4-Aminopyridine oscillations in pediatric cortical tissue are primarily mediated by activation of GABA_A_ receptors, based on electrophysiological and pharmacological observations. In whole-cell current clamp mode (K-gluconate in the patch pipette), 4-AP oscillations in CPNs were mostly depolarizing at RMP (usually around −70 mV). However, when the membrane was more depolarized (around −54 mV or less), the oscillation became hyperpolarizing. The estimated reversal potential occurred at approximately −57 mV, which corresponds to the chloride equilibrium potential in our recording conditions. This observation was confirmed in voltage clamp recordings. At a holding potential of + 10 mV, the 4-AP oscillations in CPNs (including normal-appearing, cytomegalic, and immature) were manifested as large outward currents (mean = 904.3 ± 49 pA, *n* = 120). No statistically significant difference in amplitude was observed among pathologies (*p* = 0.8, one-way ANOVA). The amplitude of the oscillation decreased as a function of the holding potential. At −70 mV holding potential the currents were small and, in most cases, became inward (mean = −103.6 ± 12 pA, *n* = 31). The estimated reversal potential also was around −57 mV (Figure 4). This value corresponds to our previous estimates for GABA_A_ receptor-mediated responses (Cepeda et al., 2007, 2014). Further, during the 4-AP oscillation, the cell membrane input resistance decreased, probably as a result of the shunting inhibition mediated by GABA_A_ receptors.

### Effects of BIC, a GABA_A_ Receptor Antagonist, on 4-AP Oscillations and Cortical Excitability

**FIGURE 5:**
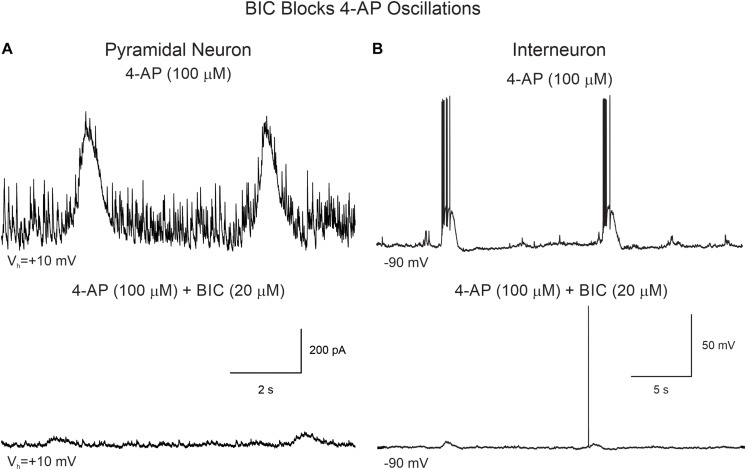
BIC reduces the amplitude of 4-AP oscillations in CPNs and interneurons. **(A)** CPN recorded in voltage clamp mode (+10 mV holding potential). After stable 4-AP oscillations were induced (upper trace), bath application of BIC (20 μM) significantly reduced the amplitude of the oscillations (lower trace). **(B)** 4-AP oscillations and bursting were induced in this interneuron recorded in current clamp mode. The cell was hyperpolarized to −90 mV by injecting negative current to prevent spontaneous firing. After BIC application, membrane depolarization amplitude and bursting were reduced.

Supporting the idea that 4-AP oscillations were mainly GABAergic and mediated by GABA_A_ receptors, the addition of BIC greatly reduced their amplitude and, in some cases, it eliminated them completely (Figure 5A). Similarly, BIC reduced the amplitude of 4-AP oscillations and bursting observed in cortical interneurons (Figure 5B).

**FIGURE 6:**
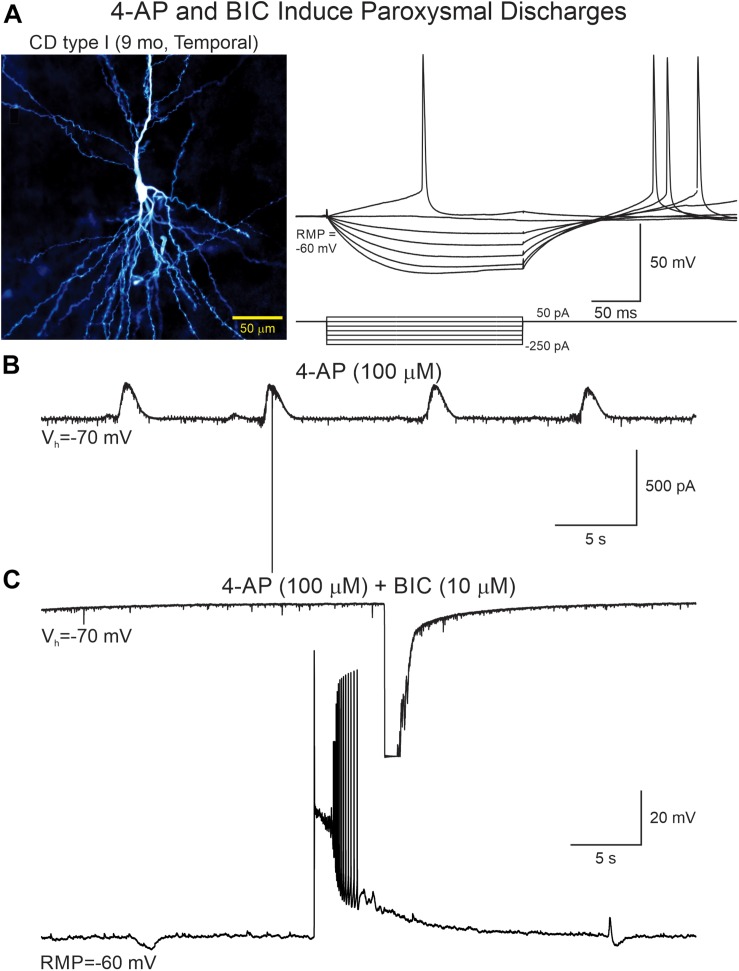
4-AP and BIC increase network excitability. **(A)** A CPN from a CD type I case was recorded in current and voltage clamp modes. Left panel shows the cell after biocytin processing. Right panel shows voltage changes induced by hyperpolarizing and depolarizing current pulses. **(B)** After addition of 4-AP rhythmic oscillations with scattered action potentials were seen. **(C)** Addition of BIC eliminated the 4-AP oscillations but simultaneously increased network excitability as demonstrated by the generation of paroxysmal discharges recorded in both voltage clamp (top trace, truncated due to saturation) and current clamp (lower trace).

An interesting observation was that in CPNs bath application of BIC reversed the polarity of the oscillations, i.e., in current clamp mode the membrane depolarization converted into a hyperpolarization (Supplementary Figure S3) and in voltage clamp (V_h_ = −70 mV) the inward current became outward. This suggested there is another minor component of the 4-AP oscillation, which is not mediated by GABA_A_ receptors. As in voltage clamp this component was outward-going, it seemed unlikely it was mediated by glutamate receptors. Further, it could occur in the presence of glutamate receptor antagonists CNQX (10 μM) and APV (50 μM). Importantly, while GABA_A_ receptor antagonists such as BIC reduced the amplitude of 4-AP oscillations, they also increased overall network excitability as demonstrated by the sporadic occurrence of paroxysmal discharges (Figure 6 and Table 2).

**TABLE 2:** Effects of 4AP + BIC.

						
			**No**	**Isolated PDs/**	**Repetitive**	**Ictal-like**
**Pathology**	**Cases**	**Cell type**	**PDs**	**bursts of APs**	**PDs**	**activity**
CD type I	7	Pyr (*n* = 10)	4	5	1	–
		Inter (*n* = 2)	1	1	–	–
CD type II	13	Pyr (*n* = 19)	7	11	1	–
		Inter (*n* = 4)	3	–	1	–
TSC	6	Pyr (n = 11)	4	7	–	–
		Inter (*n* = 0)	0			
Non-CD	5	Pyr (*n* = 11)	4	7	–	–
		Inter (*n* = 0)	0			
Total	31	Pyr (*n* = 51)	19	30	2	0
		Inter (*n* = 6)	4	1	1	0

*PD = paroxysmal discharges. AP = action potentials. Pyr = pyramidal neuron. Inter = interneuron.*

### Effects of Phaclofen, a GABA_B_ Receptor Antagonist, on 4-AP Oscillations and Cortical Excitability

**FIGURE 7:**
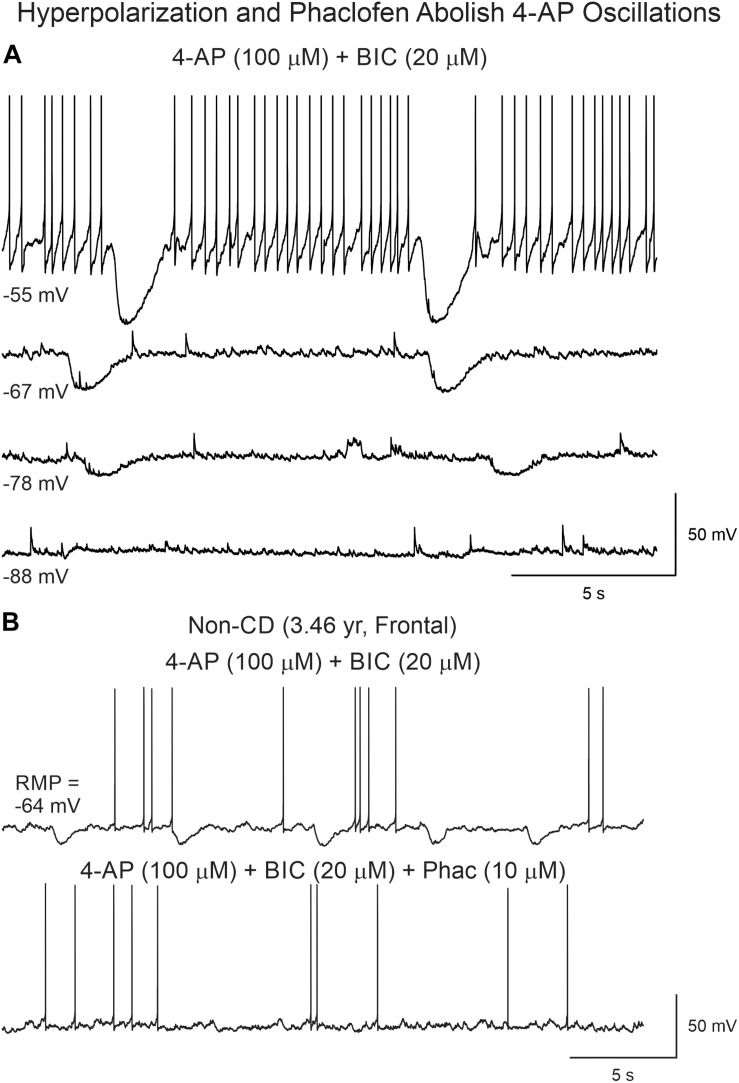
Reversal potential of 4-AP oscillations after BIC. **(A)** With increasing membrane hyperpolarization, by injection of negative current, the amplitude of the oscillation was reduced and reversed around −85 mV, suggesting involvement of K^+^ conductances, as expected if GABA_B_ receptors were activated. **(B)** Supporting this idea, addition of the GABA_B_ receptor antagonist phaclofen eliminated the 4-AP oscillation.

We attempted to determine the reversal potential of the remnant current persisting after BIC application and found that its amplitude decreased as a function of cell membrane hyperpolarization until it reversed around −85 mV (Figure 7A), strongly indicating it was mediated by K^+^ channels, in particular, inwardly rectifying K^+^ channels such as those in the Kir3 family (Mott, 2015). As GABA_B_ receptors are linked to Kir channels, this suggested that 4-AP membrane hyperpolarizations occurring after GABA_A_ receptor antagonism could be mediated by activation of GABA_B_ receptors. In support, the GABA_B_ receptor antagonist phaclofen (6–10 μM) obliterated this remnant current (Figure 7B).

In spite of inducing interictal-like membrane oscillations and enhancing overall cortical excitability, 4-AP and BIC were not sufficient, in our recording conditions, to induce ictal activity. A previous report showed that in human CD tissue GABA_B_ receptors play an important role in epileptogenesis as baclofen blocks paroxysmal discharges induced by 4-AP (D’Antuono et al., 2004). Thus, to confirm this observation, we tested the effects of a GABA_B_ antagonist, phaclofen on 4-AP oscillations. This selective GABA_B_ receptor antagonist which, as shown previously, reduced the amplitude of 4-AP oscillations, further enhanced cortical excitability and induced ictal-like activity.

**FIGURE 8:**
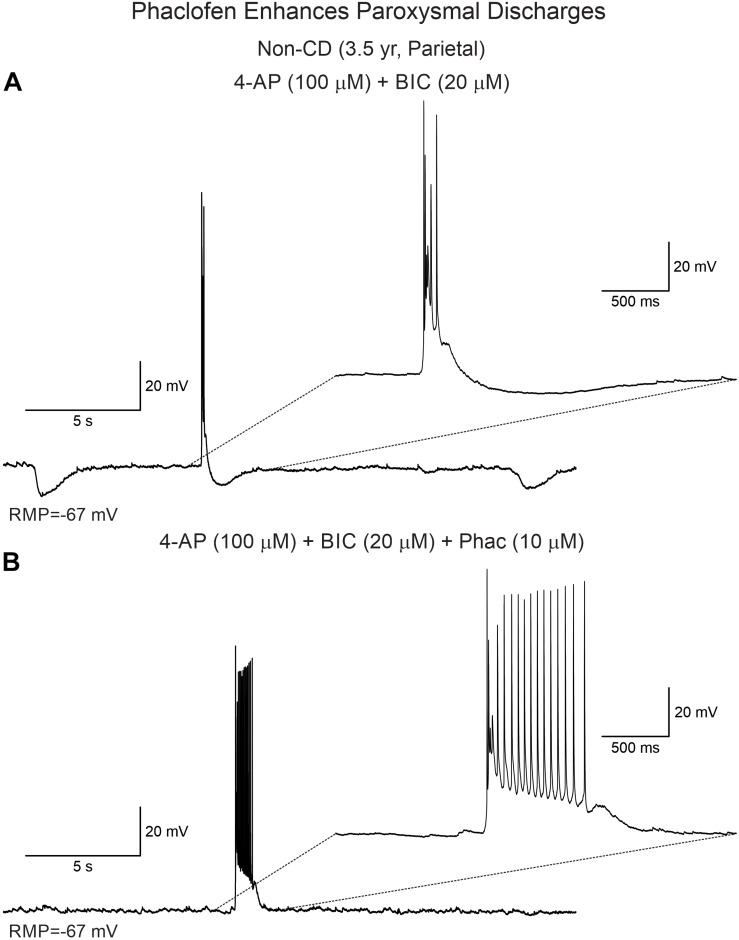
Phaclofen enhances paroxysmal activity. **(A)** In current clamp mode, paroxysmal discharges were induced by combined application of 4-AP and BIC. **(B)** After addition of phaclofen, burst duration was increased.

**FIGURE 9:**
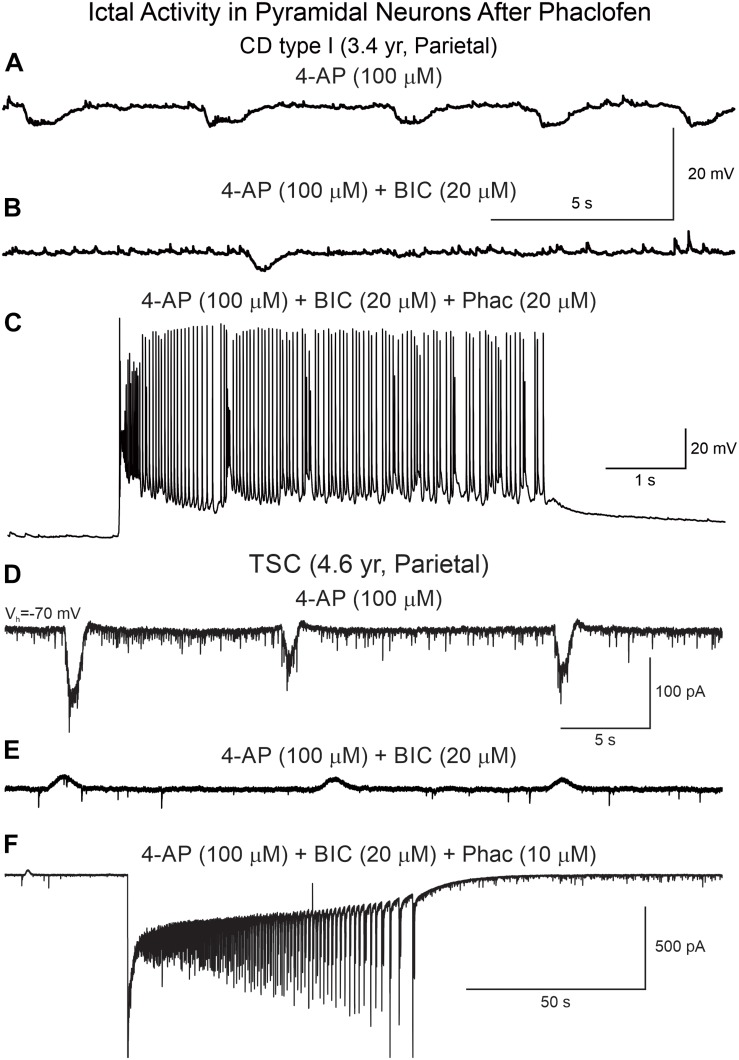
Phaclofen favors the transition to ictal-like discharges in CPNs. **(A)** Membrane oscillations were induced by 4-AP (top trace). **(B)** After BIC, the oscillations were reduced. **(C)** Addition of phaclofen to the mix generated ictal-like discharges. **(D)** In voltage clamp mode, 4-AP induced rhythmic inward currents. **(E)** After addition of BIC, the currents became outward and the amplitude was reduced. **(F)** Phaclofen in combination with 4-AP and BIC induced ictal-like activity.

**FIGURE 10:**
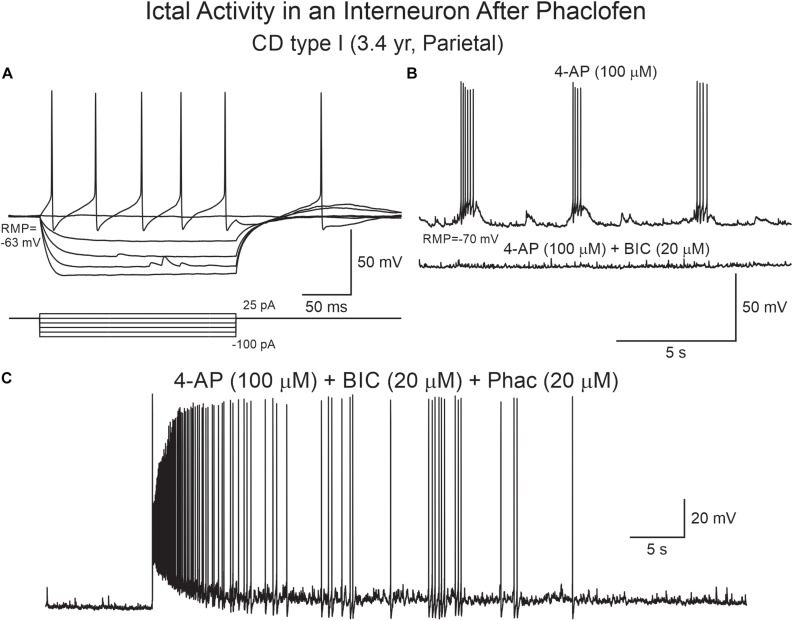
Phaclofen induces ictal-like activity in cortical interneurons. **(A)** Current clamp recording of a cortical interneuron from a CD type I case. Depolarizing current pulses induced action potentials with very large after-hyperpolarization. **(B)** After 4-AP, rhythmic depolarizations and bursting were induced. Addition of BIC almost completely eliminated bursting. **(C)** Addition of phaclofen to the mix resulted in ictal-like discharges.

Phaclofen (10–25 μM) was tested in 21 neurons (16 CPNs and five interneurons) from nine cases (three CD type I, one CD type II, four TSC, and one non-CD). At the low concentration, no enhancement of paroxysmal discharges generated by 4-AP and BIC were seen in 10 cells. In the remainder, phaclofen showed a clear proconvulsant effect. At the low concentration (10 μM), it facilitated the induction of paroxysmal discharges and/or increased burst duration (*n* = 6) (Figure 8). At the high concentration (20–25 μM), it induced ictal-like activity in three CPNs (Figure 9) and two interneurons (Figure 10). In those cells, 14 ictal-like episodes were captured and had an average duration of 21 ± 9 s (range 1.4–105 s). Ictal discharges were only seen in CD and TSC cases but not in the non-CD case. A comparison between the effects of combined drug application of 4-AP + BIC and 4-AP + BIC + Phaclofen demonstrated significantly higher epileptogenicity when the GABA_B_ receptor antagonist was present (Tables 2, 3). In 4-AP + BIC, 40.3% of neurons did not present with paroxysmal discharges (besides 4-AP oscillations), 54.4% showed isolated paroxysmal discharges or bursts of action potentials, and only 5.3% displayed repetitive paroxysmal discharges. No cells exhibited ictal-like activity. In contrast, after addition of phaclofen, all cells showed paroxysmal activity; 61.9% displayed isolated discharges or bursts of action potentials, 14.3% showed repetitive paroxysmal discharges, and 23.8% presented with ictal-like activity. The difference between groups was statistically significant (*p* < 0.001, Chi-square test).

**TABLE 3:** Effects of 4-AP + BIC + phaclofen.

			**No**	**Isolated PDs/**	**Repetitive**	**Ictal-like**
**Pathology**	**Cases**	**Cell type**	**PDs**	**bursts of APs**	**PDs**	**activity**
CD I	3	Pyr (*n* = 5)	0	4	1	–
		Inter (*n* = 2)	0	1		1
CD II	1	Pyr (*n* = 1)	0	1	–	–
		Inter (*n* = 0)	0			
TSC	4	Pyr (*n* = 8)	0	5	–	3
		Inter (*n* = 1)	0	1	–	1
Non-CD	1	Pyr (*n* = 2)	0	1	1	–
		Inter (*n* = 2)	0	0	1	–
Total	9	Pyr (*n* = 16)	0	11	2	3
		Inter (*n* = 5)	0	2	1	2

Finally, at the low concentration, phaclofen also enhanced paroxysmal discharges induced in 0 Mg^2+^ external solution in a CPN from a CD type II case (Supplementary Figure S4). This provided further proof that antagonism of GABA_B_ receptors facilitates epileptic activity, particularly in CD cases.

### Effects of eACSF (BHB and Pyruvate) on 4-AP Oscillations

It has been reported that eACSF, with BHB and pyruvate added, can reduce the depolarizing/excitatory actions of GABA in early development (Holmgren et al., 2010). In consequence, we tested the effects of eACSF on 4-AP oscillations in eight cases (*n* = 4 CD type I, 1 TSC, and 3 non-CD). Surprisingly, within minutes after changing the external solution from regular ACSF to eACSF (BHB and pyruvate), the frequency and/or amplitude of 4-AP oscillations was reduced in all, except 1, CPNs tested (*n* = 15, including three immature-looking pyramidal neurons) (Supplementary Figure S5). This suggests that addition of these energy substrates could have antiepileptic effects.

## Discussion

In the present study, we used cortical tissue samples from pediatric epilepsy surgery patients to examine CPN and interneuron sensitivity to proconvulsant drugs including 4-AP, as well as GABA_A_ and GABA_B_ receptor antagonists. 4-AP induced membrane oscillations that were mediated primarily by synaptic activity as TTX and cadmium abolished these oscillations. Cells from cases presenting with CD or TSC displayed enhanced sensitivity to proconvulsant drugs compared with non-CD cases. We also found that a subset of balloon/giant cells from CD type IIb and TSC cases can sense K^+^ surges induced by 4-AP. In addition, we demonstrate the critical and permissive role of GABA_B_ receptors in the transition to the ictal state in CD and TSC tissue, but not in non-CD tissue. These findings emphasize the importance of using *in vitro* slice preparations from human tissue to discern potential and selective new therapies in pediatric patients with intractable epilepsy from CD, TSC, and non-CD pathologies.

In slices from pediatric epilepsy cases, spontaneous interictal and/or ictal activities occur very rarely at the single-cell level, even when the sample is from the most epileptogenic area. This is probably due to the fact that tissue slices represent a reduced preparation lacking long-range excitatory inputs. However, in our hands, spontaneous paroxysmal discharges and ictal-like activity can be recorded from some GABAergic interneurons (Andre et al., 2007; Cepeda et al., 2019). In order to enhance CPN excitability, proconvulsant agents have been used traditionally. In particular, studies in human brain tissue have demonstrated the exquisite sensitivity of CD tissue samples to 4-AP, a K^+^-channel blocker that increases neurotransmitter release by prolonging action potential duration (Buckle and Haas, 1982; Avoli et al., 2003). 4-AP produces neuronal synchrony manifested by rhythmic membrane oscillations caused by synchronous GABA release from interneurons and possibly also by increases in gap junctional currents and/or permeability (Traub et al., 1995; Traub et al., 2001; Szente et al., 2002). Here, in pediatric epilepsy cases, we confirmed the principal role of GABA_A_ receptors in the induction of 4-AP oscillations, as blockade of these receptors with BIC greatly reduced their amplitude. However, network excitability was increased further as demonstrated by the occurrence of paroxysmal discharges, likely mediated by activation of glutamate receptors. Notably BIC, in combination with the GABA_B_ receptor antagonist, phaclofen abolished 4-AP oscillations while simultaneously allowing the transition from an interictal to an ictal state in both CPNs and interneurons, particularly from CD and TSC cases.

GABA_B_ receptors have been largely neglected compared to GABA_A_ receptors in the treatment of epileptic patients. Most studies on epileptogenic mechanisms have concentrated on studying the role of GABA_A_ receptors due to their ability to produce fast inhibition of excitatory neurons. Their potential to induce excitatory effects has also been examined profusely in developing brains (Ben-Ari, 2014, 2015). In contrast to GABA_A_ receptors, which can mediate depolarizing and potentially excitatory actions, activation of postsynaptic GABA_B_ receptors is always inhibitory. However, studies on the role of GABA_B_ receptors in epileptogenesis are much more limited but their importance is becoming more and more recognized (Craig and McBain, 2014). The present study in pediatric epilepsy surgery patients confirms experimental data showing that GABA_B_ receptors play a key role in the transition from interictal to ictal activity (Swartzwelder et al., 1987; Watts and Jefferys, 1993; Scanziani et al., 1994). They are also in line with multitude experimental studies demonstrating that antagonism of GABA_B_ receptors is epileptogenic (Badran et al., 1997; Sutor and Luhmann, 1998; Prosser et al., 2001; Motalli et al., 2002; Uusisaari et al., 2002). In another study, it was shown that GABA_B_ receptors also control the depolarizing response mediated by GABA_A_ receptors as blocking GABA_B_ receptors makes this depolarization excitatory and proconvulsant (Kantrowitz et al., 2005).

Another novel finding was that balloon/giant cells can also display membrane oscillations, suggesting that these undifferentiated cells, with variable expression of glial and neuronal markers (Cepeda et al., 2003; Jozwiak et al., 2006; Blumcke et al., 2011), can sense K^+^ accumulations. The oscillations in balloon/giant cells were significantly smaller and slower than those in CPNs and interneurons. At present, it remains unknown what the function of these oscillations in balloon/giant cells might be. However, based on the fact that these cells share more resemblance to astrocytes than neurons (Cepeda et al., 2006), it can be speculated that their function is to buffer K^+^ surges to prevent the occurrence of epileptic discharges. Interestingly, it has been reported that in CD type IIb, areas with balloon cells are less epileptogenic than adjacent cortex (Boonyapisit et al., 2003). Further, while ictal activity can be generated in areas with histopathologic CD type IIa, areas with CD type IIb do not show seizure activity, suggesting a possible protective role of balloon cells (Boonyapisit et al., 2003).

While multitude studies have emphasized that GABA interneuron synchrony jump starts focal seizures (Avoli et al., 1999, 2003; D’Antuono et al., 2004; Shiri et al., 2015; de Curtis and Avoli, 2016; Blauwblomme et al., 2018; Elahian et al., 2018), the respective role of GABA_A_ and GABA_B_ receptors has not been elucidated. Based on our observations, we can propose that relaxation of GABA inhibition mediated by GABA_B_ receptors is responsible for the transition to ictal activity. In a previous study using CD tissue from mostly teenage and adult patients, D’Antuono et al. (2004) demonstrated that baclofen, a GABA_B_ receptor agonist, stopped seizure activity induced by 4-AP and proposed that these receptors can be a target of AEDs. They also concluded that GABA_A_ receptors lead to network synchrony and generation of ictal activity. While here we confirmed that GABA_B_ receptors play a critical role in the modulation of seizure activity, our study also showed that GABA_A_ receptors are not an absolute requirement for seizure generation since ictal activity was observed even after full blockade of these receptors with BIC.

What makes CD tissue particularly susceptible to proconvulsant drugs? The etiology, age of onset, and pathological substrates of pediatric epilepsy are very diverse. Thus, the sensitivity to proconvulsant and AEDs could be different depending on age and pathology. The most common substrate in epilepsy surgery patients less than 5 years of age is CD, a malformation characterized by architectural abnormalities (CD type I) of the cerebral cortex and, in severe cases, the presence of large, dysmorphic neurons and balloon cells (CD type IIa/b). The histopathology of TSC is very similar to that observed in CD type IIb. Interestingly, in pediatric epilepsy cases, GABA synaptic activity is not reduced. On the contrary, we demonstrated that GABA_A_ receptor-mediated synaptic activity is increased compared with glutamatergic activity (Cepeda et al., 2005b) and in some cases GABA is depolarizing (Cepeda et al., 2007). More recently, we found that areas with pathological high-frequency oscillations (HFOs) had a significant increase in spontaneous GABA synaptic activity as well as pacemaker GABA synaptic activity (PGA) (Cepeda et al., 2014, 2019). In light of the present results, it can be speculated that such an increase can be the result of enhanced GABAergic interneuron excitability and dysfunction of presynaptic GABA_B_ receptors (D’Antuono et al., 2004). Importantly, GABA_B_ receptors are also present postsynaptically on CPNs, are always inhibitory *via* activation of inwardly rectifying K^+^ channels as well as inhibition of voltage-gated Ca^2+^ channels, and could dampen CPN excitability.

We also found that addition of a ketone body, BHB, reduced 4-AP oscillations and, presumably, cortical hyperexcitability. In pediatric epilepsy, the ketogenic diet has demonstrated beneficial effects. However, the antiepileptic mechanisms of this diet are multiple and still remain ill-defined (Rho, 2017; Simeone et al., 2017). Importantly, experimental studies have demonstrated that the ketone body BHB reduces seizure-like activity in a drosophila melanogaster model (Li et al., 2017). In the same study, it was shown that a K_ATP_ blocker or a GABA_B_ receptor antagonist (CGP-55845) reversed BHB effects, providing conclusive evidence that the beneficial effects of the ketogenic diet are likely mediated by K_ATP_ channels and GABA_B_ receptors. In pediatric epilepsy, a prospective study found a positive and strong correlation between measures of seizure frequency and BHB blood concentrations. Although the results did not reach statistical significance, likely due to the relatively small number of cases, a larger study is warranted (Buchhalter et al., 2017).

## Limitations

We acknowledge that the present study has a number of important limitations. First and foremost, all patients were taking AEDs, some of which directly affect GABA transmission and potentially GABA_A/B_ receptor function. Second, studies have shown that the slice preparation alters chloride concentrations due to trauma caused by the slicing procedure, so that more superficial cells have increased intracellular chloride and, in consequence, a more depolarized reversal potential (Dzhala et al., 2012). Third, our recordings were obtained at room temperature which, although helping preserve tissue integrity, it also decreases overall neuronal excitability (Javedan et al., 2002). Fourth, the GABA_A_ receptor antagonist we used (BIC) also blocks the small Ca^2+^-activated K^+^ currents (Khawaled et al., 1999), which could further enhance neuronal excitability. Finally, in some experiments testing the GABA_B_ receptor antagonist, phaclofen, we used a Cs-based internal solution. Cs^+^ is known to reduce inwardly rectifying K^+^ currents, which could have contributed to some of the findings. However, the same observations were confirmed using K-gluconate as the internal solution. Thus, in spite of these limitations, our work allows to reach several important conclusions.

## Conclusion and Clinical Implications

In conclusion, using the 4-AP model of epileptogenesis in combination with GABA_A/B_ receptor antagonists, we demonstrate a critical role of GABA_B_ receptors in the transition from interictal to ictal activity. When GABA_B_ receptors are functional, they are able to prevent catastrophic excitation of CPNs. However, when they are disabled, ictal activity is facilitated. This implies that ensuring proper function of GABA_B_ receptors is critical for keeping a normal balance between excitation and inhibition. Use of allosteric modulators of GABA_B_ receptors at postsynaptic sites could hold promise as effective antiepileptic agents in cases of pediatric epilepsy not responsive to common AEDs (Morrisett et al., 1993; Ong and Kerr, 2005; Mares, 2012; Lang et al., 2014).

## Data Availability Statement

The data resulting in this publication are available from the corresponding author upon reasonable request and provided patient confidentiality is preserved.

## Ethics Statement

The present study was approved by the IRB at UCLA (No. 11-000030-CR-00009). Parents or responsible persons signed written informed consents to allow use of pathological tissue samples for research purposes. No tissue was resected outside the area deemed affected by the pathology and required for seizure control.

## Author Contributions

GM, ML, and CC designed the study. GM resected the tissue samples. CC and JB performed slice recordings. CT, BV, SL, JB, and CC analyzed the electrophysiological data and prepared the figures. HV examined the pathological tissue samples and identified the histopathology. SL and CC co-wrote the manuscript. All the authors contributed to the final edition.

## Conflict of Interest

The authors declare that the research was conducted in the absence of any commercial or financial relationships that could be construed as a potential conflict of interest.

## Supplementary Material

FIGURE S1 Tetrodotoxin (TTX) and cadmium block 4-AP oscillations. **(A)** Voltage clamp recording (holding potential, Vh = +10 mV) of a pyramidal neuron from a CD type IIb case. 4-AP oscillations were completely blocked after 3 min bath application of TTX. **(B)** Current clamp recording (at RMP) of a pyramidal neuron from a CD type I case. Cadmium blocked 4-AP oscillations after 3 min bath application.

FIGURE S2 4-AP oscillations in balloon cells. **(A)** A balloon cell from a CD type IIb case was recorded and filled with biocytin. **(B)** Depolarizing current pulses were unable to induce action potentials (top right panel). However, bath application of 4-AP induced slow, small amplitude membrane depolarizations (bottom right panel), suggesting they might be sensing increases in K^+^ concentration.

FIGURE S3 BIC inverts the polarity of 4-AP oscillations. **(A)** In the presence of BIC, 4-AP oscillations were depolarizing and accompanied by action potentials. After addition of BIC to the bath solution, the oscillations reversed polarity and became hyperpolarizing. **(B)** In this CPN 4-AP oscillations were biphasic (hyperpolarization followed by depolarization). After BIC, the oscillation was purely hyperpolarizing and inhibitory at two membrane potentials. Notice that the action potentials in the upper panels are truncated.

FIGURE S4 Phaclofen enhances paroxysmal discharges in the zero Mg^2+^ model. **(A)** In this CPN from a CD type II case, removal of Mg^2+^ from the external solution increased cell excitability as reflected by spontaneous firing. **(B)** Addition of BIC induced rhythmic paroxysmal discharges. **(C)** After phaclofen burst duration was significantly increased.

FIGURE S5 Enriched ACSF (BHB and pyruvate) blocks 4-AP oscillations. **(A)** A CPN was recorded and filled with biocytin. **(B)** In voltage clamp mode, at a +10 mV holding potential, spontaneous GABA synaptic activity was observed. After 4-AP, rhythmic oscillations (outward currents) were induced. A few minutes after switching the bathing solution to eACSF, 4-AP oscillations were abated. After wash out in normal ACSF, some 4-AP oscillations returned.
