# Supplementary figures and images for: Paroxysmal Discharges in Tissue Slices From Pediatric Epilepsy Surgery Patients: Critical Role of GABAB Receptors in the Generation of Ictal Activity
Source: Front Cell Neurosci. 2020 Mar 20;14:54. doi: 10.3389/fncel.2020.00054 (PMC7099654; doi:10.3389/fncel.2020.00054)

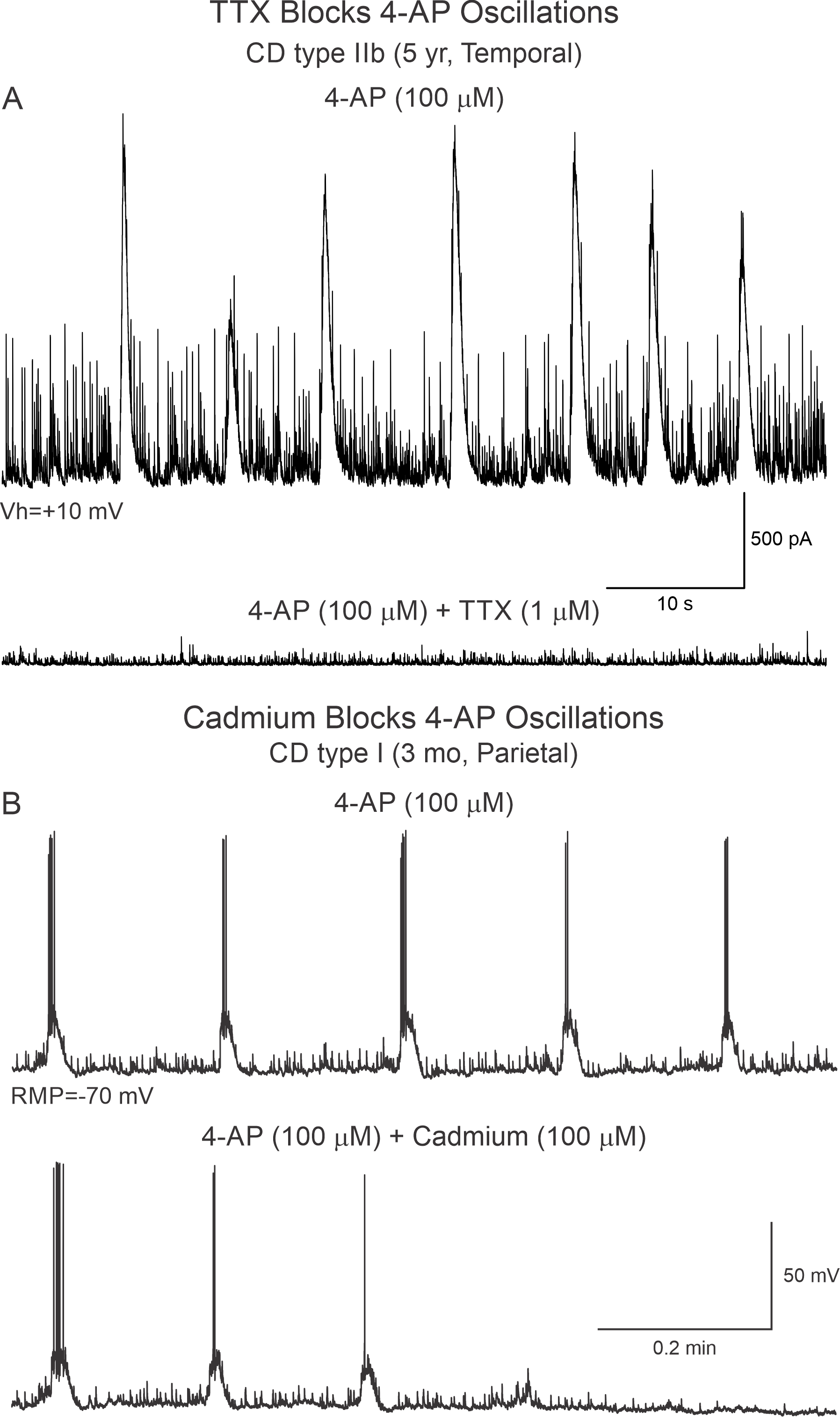

Supplement: FIGURE S1 — Tetrodotoxin (TTX) and cadmium block 4-AP oscillations. (A) Voltage clamp recording (holding potential, Vh = +10 mV) of a pyramidal neuron from a CD type IIb case. 4-AP oscillations were completely blocked after 3 min bath application of TTX. (B) Current clamp recording (at RMP) of a pyramidal neuron from a CD type I case. Cadmium blocked 4-AP oscillations after 3 min bath application. [file Image_1.tif]
